# Pre‐hospital transfusion of red blood cells. Part 1: A scoping review of current practice and transfusion triggers

**DOI:** 10.1111/tme.12667

**Published:** 2020-02-21

**Authors:** Elisabeth C. van Turenhout, Sebastiaan M. Bossers, Stephan A. Loer, Georgios F. Giannakopoulos, Lothar A. Schwarte, Patrick Schober

**Affiliations:** ^1^ Department of Anaesthesiology Amsterdam UMC, Vrije Universiteit Amsterdam Amsterdam The Netherlands; ^2^ Department of Trauma Surgery Amsterdam UMC, Vrije Universiteit Amsterdam Amsterdam The Netherlands; ^3^ Helicopter Emergency Medical Service “Lifeliner 1” Amsterdam UMC, Vrije Universiteit Amsterdam Amsterdam The Netherlands

**Keywords:** damage control resuscitation, emergency medical service, major haemorrhage, pre‐hospital transfusion, red blood cells, transfusion criteria

## Abstract

**Objectives:**

The primary aim of this scoping review is to describe the current use of pre‐hospital transfusion of red blood cells (PHTRBC) and to evaluate criteria used to initiate PHTRBC. The effects on patients' outcomes will be reviewed in Part 2.

**Background:**

Haemorrhage is a preventable cause of death in trauma patients, and transfusion of red blood cells is increasingly used by Emergency Medical Services (EMS) for damage control resuscitation. However, there are no guidelines and little consensus on when to initiate PHTRBC.

**Methods:**

PubMed and Web of Science were searched through January 2019; 71 articles were included.

**Results:**

Transfusion triggers vary widely and involve vital signs, clinical signs of poor tissue perfusion, point of care measurements and pre‐hospital ultrasound imaging. In particular, hypotension (most often defined as systolic blood pressure ≤ 90 mmHg), tachycardia (most often defined as heart rate ≥ 120/min), clinical signs of poor perfusion (eg, prolonged capillary refill time or changes in mental status) and injury type (ie, penetrating wounds) are common pre‐hospital transfusion triggers.

**Conclusions:**

PHTRBC is increasingly used by Emergency Medical Services, but guidelines on when to initiate transfusion are lacking. We identified the most commonly used transfusion criteria, and these findings may provide the basis for consensus‐based pre‐hospital transfusion protocols.

## 1 INTRODUCTION

Trauma is a major cause of death and disability.^1^, ^2^ In injured patients, death within 24 hours is primarily attributable to haemorrhage,^3^, ^4^, ^5^ and many of these deaths are potentially preventable.^6^, ^7^, ^8^ To optimise bleeding patients' odds of survival, pre‐hospital treatment focuses on early haemorrhage control to avoid hypovolaemic shock and the lethal triad of acidosis, hypothermia and coagulopathy. This is commonly attempted with topical treatment,^9^ such as haemostatic dressings, tourniquets and/or pelvic binders. However, haemorrhage is often non‐compressible and cannot be controlled by topical treatment alone.^7^, ^10^, ^11^ Moreover, patients may already be in haemorrhagic shock when pre‐hospital healthcare providers arrive at a scene. Therefore, to replace (ongoing) blood loss, liberal volume replacement, typically with readily available crystalloid fluids, has been long advocated.^12^ However, this practice is associated with adverse effects including dilutional coagulopathy, acidosis, hypothermia and accelerated blood loss.^13^ In contrast, damage control resuscitation (DCR) involves restrictive fluid resuscitation, which avoids crystalloids while accepting some degree of hypotension, and is increasingly preferred until bleeding can be surgically controlled.^13^, ^14^, ^15^, ^16^, ^17^, ^18^

Red blood cells (RBCs) provide a more effective volume expansion than crystalloids; the infusion of large volumes of crystalloids or colloids can thus be avoided. The RBCs benefit haemostasis and thrombosis^19^ and restore oxygen‐carrying capacity,^20^ thereby potentially reducing acidosis through tissue hypoxia. Military medical teams have long been transfusing blood products prior to patients' arrival at a surgical unit.^4^, ^21^ As proposed by Jansen et al,^22^ differences in survival between civilian casualties who require massive transfusion (60%)^23^ and military casualties (93%)^22^ may be partly explained by this practice.

In recent years, civilian Emergency Medical Services (EMS) are increasingly transfusing RBCs before hospital arrival.^24^, ^25^ However, logistic and operational challenges are hampering the widespread implementation of blood transfusions in the pre‐hospital setting. Moreover, consensus regarding pre‐hospital indications for blood transfusions is lacking, and evidence regarding the efficacy of this practice is scarce. Therefore, this systematic review consists of two parts. Part 1 is a scoping review in which we systematically gathered the research done in the area of pre‐hospital transfusion of red blood cells (PHTRBC), aiming to describe the current challenges of pre‐hospital transfusion and, in particular, to evaluate which criteria are currently used to initiate PHTRBC. This review may serve as guide to derive consensus based pre‐hospital transfusion protocols and informed practice guidelines. In Part 2, the effect on patient outcomes will be systematically appraised.

## 2 METHODS

The review was registered at Prospero (website: https://www.crd.york.ac.uk/prospero, identification number: CRD42018084658) and was conducted in accordance with PRISMA‐Scr (Preferred Reporting Items for Systematic Reviews and Meta‐Analyses—Extension for Scoping Reviews) guidelines.^26^

### 2.1 *Information sources, search strategy and study selection*

PubMed and Web of Science were searched through January 2019 using the following terms: PUBMED: (“emergency medical services”[MeSH Terms] OR (“emergency”[All Fields] AND “medical”[All Fields] AND “services”[All Fields]) OR “emergency medical services”[All Fields] OR “Warfare”[Mesh] OR “Warfare”[All Fields] OR combat[All Fields]) AND (“blood transfusion”[MeSH Terms] OR (“blood”[All Fields] AND “transfusion”[All Fields]) OR “blood transfusion”[All Fields]). WEB OF SCIENCE: TOPIC: (((emergency medical services) OR (emergency AND medical AND services) OR (emergency medical services) OR (Warfare) OR (combat)) AND ((blood transfusion) OR (blood AND transfusion))). Titles and abstracts were screened by two reviewers, and the full texts of all potentially eligible articles were retrieved.

All manuscripts discussing PHTRBC were eligible for inclusion, provided they were written in English, German, French or Dutch. Since we sought to describe typical practice and identify commonly used indications and transfusion triggers, we did not limit the inclusion to studies allowing comparisons between PHTRBC patients and controls. Reviews and editorials were excluded. Eligibility was assessed independently in a blinded manner by two reviewers (ET & SB). Disagreements about manuscript eligibility were resolved by discussion within the investigator group. Reference lists of suitable articles were screened for additional relevant content.

### 2.2 *Data abstraction*

We developed a standardised data‐extraction sheet, which was refined after testing with the first 20 articles. In one case, further information was obtained after contacting the authors.^27^ The setting and type of transport the EMS used (civilian or military, scene or interfacility), as well as the availability, frequency and volume of PHTRBC transfusions were extracted. Descriptions of problems that arose during PHTRBC are summarised in the text.

Transfusion criteria were recorded and classified as “major criteria” or “minor criteria,” depending on whether only one criterion needed to be met to initiate PHTRBC or whether a combination of several criteria was required. Some groups published studies regarding pre‐hospital transfusion with the same EMS more than once. In this case, only the most recent and best specified description of transfusion criteria was considered. Criteria are summarised in diagrams (Figures 2 and 3).

## 3 RESULTS

### 3.1 *Selection of articles*

**Figure 1:**
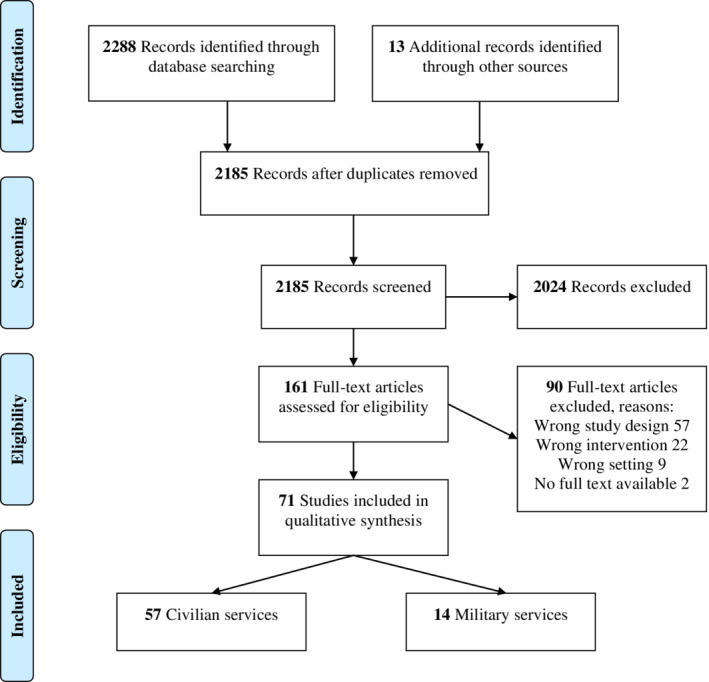
PRISMA flow diagram

The search in PubMed and Web of Science yielded 2172 hits after removal of duplicates. Of which, 2024 articles were excluded based on the title and/or abstract, because they did not discuss PHTRBC or were not original research (eg, editorials, reviews). Ninety articles were excluded after screening their full texts because they did not meet the inclusion criteria. For this review, 71 articles were included (Figure 1).

In total, 57 articles discussed PHTRBC in civilian medical services. Notably, five articles primarily dealt with a different study topic, but were included as they additionally provided valuable information regarding PHTRBC.^28^, ^29^, ^30^, ^31^, ^32^ Table 1 describes studies' characteristics, listing study design, region and period in which the study took place, the primary goal, study group and control group, whether matching occurred, the number of subjects, the type of transport and, in the case of trauma patients, the mechanism of injury and Injury Severity Score.

**Table 1:** Overview of studies

References	Region	Study period	Primary goal	Study group	Control group	Control for confounding	Patients transfused (n)^a^	Type of transport (%trauma)	Mechanism of injury	ISS
*Civilian services*										
Prospective comparative studies										
Henriksen^66^	Texas, United States	2012‐2013	To investigate the association between PHTRBC and PHT‐plasma and haemostatic function	Receivers of PHTRBC and/or PHT‐plasma	Receivers of in‐hospital transfusion	Adjusted data	75^b^	Scene (100%)	PHT: Blunt: 55% Penetrating: 45% Control: Blunt: 75% Penetrating: 25% *P* = .002	PHT: 29 (17‐41) Control: 26 (17‐34) *P* = .106
Holcomb^52^	United States (9 trauma centres)	2015	To study the effect of PHTRBC and/or PHT‐plasma on in‐hospital mortality	Severely injured receivers of PHTRBC and/or plasma	No pre‐hospital blood products	Propensity score	142^b^	Scene (100%)	PHT: Blunt: 79.1% Penetrating: 20.9% Matched control: Blunt: 72.7% Penetrating: 27.3%	PHT: 24 (10‐34) Control: 22 (10‐34)
Smith^82^	Midlands East of England, United Kingdom	2016‐2020	Study protocol for RCT: to investigate the effect of PHTRBC and PHT‐plasma on tissue perfusion and mortality	Receivers of PHTRBC and/or PHT‐plasma	Receivers of crystalloids	RCT	Plan: 490	n/d (100%)	n/d	n/d
Retrospective comparative studies										
Brown^48^	United States (9 institutions)	2003‐2010	To characterise the association of pre‐trauma centre RBC with mortality and TIC in severely injured patients with blunt trauma	Receivers of pre‐trauma centre RBC	No pre‐hospital transfusion	Propensity score	50	Scene+ interfacility (100%)	Blunt: 100% Penetrating: 0% (per exclusion)	PHTRBC: 34 (18‐43) Control: 30 (23‐43) *P* = .81
Brown^49^	Pennsylvania, United States	2007‐2012	To evaluate the association of pre‐trauma centre RBC with outcomes	Receivers of pre‐trauma centre RBC	No pre‐hospital transfusion	Propensity score	240 matched (71 scene)	Scene+ interfacility (100%)	PHTRBC: Blunt: 191 (80%) Penetrating: 49 (20%) Matched controls: Blunt: 395 (82%) Penetrating: 85 (18%)	PHTRBC: 18 (10‐29) Matched control: 17 (9–27) *P* = .05
Griggs^69^	Kent, Surrey and Sussex, United Kingdom	2010‐2015	To compare mortality for patients with suspected traumatic haemorrhage receiving PHTRBC compared with crystalloid	Code Red patients receiving PHTRBC	Code Red patients receiving crystalloids	Adjusted data	92	Scene (100%)	PHTRBC: Blunt: 95% Penetrating: 5% MVC 68% Fall 9% Control: Blunt: 99% Penetrating: 1% MVC: 58% Fall: 9%	Mean (SD) PHTRBC:32 (12) Control: 21 (14) *P* = .67
Holcomb^25^	Texas, United States	2011‐2013	To evaluate the effect of PHTRBC and/or PHT‐plasma on survival and blood product use	Receivers of PHTRBC and/or PHT‐plasma	Receivers of in‐hospital transfusion	Adjusted data	137^b^	Scene (100%)	PHT: Blunt: 77% Penetrating: 23% Control: Blunt 83% Penetrating 17% *P* = .447	PHT: 22 (12‐29) Control: 22 (11‐33) *P* = .998
Kim^29^	Minnesota, United States	2009‐2011	To analyse the effect of PHT‐plasma on coagulopathy	Receivers of PHT‐plasma + PHTRBC	Receivers of PHTRBC only	No	59 (of whom 50 RBC only)	Scene + interfacility (100%)	Plasma: Blunt: 67% Penetrating: 33% PHTRBC only: Blunt: 82% Penetrating 18% *P* = .317	Plasma: 27 PHTRBC: 23 *P* = .918
Miller^50^	Tennessee, United States	2007‐2013	To examine the impact of PHTRBC on mortality	Receivers of PHTRBC	No pre‐hospital transfusion	Propensity score	231 (195 matched)	Scene (100%)	PHTRBC: Blunt: 78% Penetrating: 22% Matched control: Blunt: 90% Penetrating: 10% *P* < .001	PHTRBC: 34 (22‐43) Matched control: 32 (22‐43) *P* = .903
Parker^67^	Minnesota, United States	2010‐2014	To examine PHT of plasma and/or RBC on outcomes in exsanguinating GI bleeding	Receivers of PHTRBC and/or PHT‐plasma with acute GI bleeding	Against GI‐bleed patients without transfusion	No	112^b^	Interfacility (0%)	n/a	n/a
Peters^74^	Nijmegen and Rotterdam, The Netherlands	2007‐2015	To establish the efficacy and safety of the PHTRBC by HEMS	Receivers of PHTRBC	Receivers of crystalloids only	Matched	73 (50 matched)	Scene (100%)	PHTRBC: Blunt: 93% Penetrating: 7% MVC 70% Fall from height 10% Matched control: Blunt: 94% Penetrating: 6% MVC 68% Fall from height 12%	PHTRBC: 34 (9‐75) Control: 35 (18‐75) *P* = .242
Price^65^	Oregon, United States	1989‐1995	To evaluate the efficacy of early blood transfusion	Receivers of PHTRBC during air transport	Receivers of crystalloids in ground transport	Matched	84	n/d (100%)	n/d	n/d
Rehn^63^	London, United Kingdom	2009‐2015	To investigate the effect of PHTRBC on overall blood product use	“Code Red” patients after implementation of PHTRBC	“Code Red” patients before implementation of PHTRBC	Adjusted data	128	Scene (100%)	PHTRBC: Blunt: 64.8% Penetrating: 35.2% MVC: 42.2% Falls: 11.7% Control: Blunt: 68.6% Penetrating: 31.4% MVC: 42.3% Falls: 12.4% Other blunt: 13.9%	PHTRBC: 29 (25‐43) Control: 27 (19‐41)
Rehn^51^	London, United Kingdom	2009‐2015	To investigate the effect of PHTRBC on mortality	“Code Red” patients after implementation of PHTRBC	“Code Red” patients before implementation of PHTRBC	Adjusted data	239	Scene (100%)	PHTRBC: Blunt: 146 (61%) Penetrating 93 (39%) Control: Blunt: 189 (63%) Penetrating: 111 (37%)	n/d
Sumida^73^	Tennessee and Connecticut, United States	1995‐1996	To analyse the effect of PHTRBC on physiologic parameters and outcome	Receivers of PHTRBC	Receivers of crystalloids only	No	17	Scene + interfacility (100%)	n/d	PHTRBC 28 Control: 27.8 *P* = .957
Prospective not‐comparative studies										
Chang^32^	United States (9 trauma centres)	2015	To describe the phenotype and laboratory coagulation abnormalities of clinically evident coagulopathic bleeding (CC) after trauma	Highest‐risk trauma patients, CC+	CC−	Adjusted data	PHTRBC in CC+ vs CC− 18 (44%) vs 82 (8%) *P* < .001	Scene (100%)	Overall: CC+ vs CC−: Blunt: 28 (68%) vs 792 (81%) Penetrating: 12 (30%) vs 165 (17%) Both: 1 (2%) vs 21 (2%) Injury type *P* = .09	CC+: 32 (25‐41) CC−: 17 (8‐27) *P* < .001
Reed^31^	Scotland	2013‐2015	To evaluate the pre‐hospital activation of Code Red	Patients for whom a pre‐hospital Code Red was activated	None	n/a	16	n/d (100%)	Overall: Blunt: 44 (83%) Penetrating: 9 (17%)	Overall: 24 (14‐37)
Sherren^72^	Greater Sydney Area, Australia	n/s (5 years.)	To describe PHTRBC	Missions involving PHTRBC	None	n/a	147	n/d (100%)	Blunt: 93.9% Penetrating: 6.1% MVC: 79 Fall from height: 3.4% Other: 11.6%	RTS: 5.967 (4.083‐6.904)
Tilney^43^	New England, United States	n/s (7 years.)	To examine utilisation, indications and outcomes in PHTRBC	Receivers of PHTRBC	None	n/a	179	Scene + interfacility (70%)	n/d	n/d
Weaver^71^	London, United Kingdom	2012	To examine the impact of on‐scene PHTRBC for seriously injured patients	Receivers of PHTRBC	None	n/a	50	Scene (100%)	n/d	n/d
Retrospective not‐comparative studies										
Bamber^60^	East of England, United Kingdom	2013	To determine how blood was transferred with patients and the fate of this blood	RBCs and/or FFP transferred with patients	None	n/a	PHTRBC: 4	Interfacility (in PHTRBC 75%)	n/d	n/d
Berns^42^	Minnesota, United States	1993‐1996	To document the development of protocols for and to review the experience with PHTRBC	Receivers of PHTRBC	None	n/a	94	Scene + interfacility (48%)	n/d	n/d
Bodnar^68^	Greater Brisbane, Australia	2011‐2012	To describe the characteristics, clinical interventions and the outcomes of PHTRBC patients	Receivers of PHTRBC	None	n/a	71	Scene (100%)	Blunt: 73.2% Penetrating: 26.8% MVC 67%	Mean (SD) 32.1 (18.2)
Dalton^64^	Oregon Washington, United States	1985‐1992	To show that PHTRBC is safe and practical	Receivers of PHTRBC with MAST	Receivers of PHTRBC without MAST	n/a	112	n/d (100%)	Overall: Blunt: 86% Penetrating: 14% MVC: 72%	Mean: MAST: 33 Non‐MAST: 31
Fahy^53^	Minnesota, United States	2002‐2014	To report our experience with a pre‐hospital transfusion protocol in paediatric patients	Paediatric trauma patients receiving PHTRBC and/or PHT‐plasma	Paediatric non‐trauma patients receiving PHTRBC and/or PHT‐plasma	n/a	28^b^	Scene+ interfacility (57%)	Blunt: 88% Penetrating: 12% MVC: 63% Gunshot wounds: 13%	24 (range 9‐66)
Heschl^61^	Victoria, Australia	2011‐2015	To describe the characteristics of PHTRBC	All cases where approval for PHTRBC was sought by paramedics	None	n/a	142	Scene (96%)	Blunt/penetrating: n/d MVC: 88% Crush/fall/other: 11.8%	Mean (SD): 36.5 (15.8)
Higgins^45^	Maine, United States	2007‐2008	To describe PHTRBC with respect to safety and efficacy and improvement in condition	Receivers of PHTRBC	None	n/a	45	Scene+ interfacility (71%)	n/d	n/d
Hooper^4^	Southwest United Kingdom	2015‐2016	To describe experience with PHTRBC	Receivers of PHTRBC	None	n/a	62	n/d (84%)	n/d	n/d
Krook^44^	Western Canada	2013‐2017	To describe the implementation and stewardship of a pre‐hospital blood transfusion programme	Receivers of PHTRBC	None	n/a	274	Scene + interfacility (74%)	n/d	n/d
Krugh^41^	Ohio, United States	1991‐1993	To describe and review the implementation of an off‐site blood product storage programme	Receivers of PHTRBC	None	n/a	8	n/d (50%)	n/d	n/d
Lyon^70^	Kent, Surrey and Sussex, United Kingdom	2013‐2014	To describe the characteristics of receivers of PHTRBC and evaluate their subsequent in‐hospital needs	Receivers of PHTRBC	None	n/a	147	Scene (97%)	Blunt: 128 (87%) Penetrating: 14 (10%) MVC: 103 (73%) Fall from height: 17 (11.6%)	33 (SD 13.4)
Maher^58^	Washington, United States	2015	To describe the development of a HEMS transfusion programme	Receivers of PHTRBC or PHT‐plasma	None	n/a	RBC 13 FFP 3	Scene + interfacility (85%)	n/d	n/d
Mena‐Munoz^46^	Pennsylvania, Ohio, and Maryland, United States	2003‐2012	To characterise receivers of out of hospital transfusion of blood products (mostly RBCs and/or plasma) during critical care transport	Receivers of out of hospital blood products	None	n/a	1440^b^	Scene + interfacility (19%)	n/d	n/d
Mix^79^	Minnesota, United States	2011–2015	To determine whether opportunities for blood product administration by ground ALS ambulances exist	Adult trauma patients with potential need for pre‐hospital blood administration	None	n/a	28	Scene (100%)	Blunt: 26 (93%) Penetrating: 2 (7%)	n/d
Moylan^28^	North Carolina, United States	1985	To analyse the effect of air vs ground inter‐hospital transport on survival	Patients with trauma scores ≤12 transported by helicopter	Patients with trauma scores ≤12 transported by ground	Matched	50	Scene + interfacility (100%)	Ground: MVC/industrial accident: 76% Assault: 24% Air: MVC or industrial accident: 85% Assault: 15%	n/d
Potter^54^	Minnesota, United States	2003–2012	To summarise our initial experience with PHTRBC and PHT‐plasma in paediatric trauma patients	Receivers (< 18 years) of PHTRBC and/or PHT‐plasma	None	n/a	16^b^	Scene + interfacility (100%)	Blunt: 87.5% Penetrating: 12.5%	Mean 30 (range 9–66)
Powell	Ohio, United States	2010‐2013	To evaluate the influence of the time between injury and transfusion on outcome	Receivers of RBC within 24 hours of hospital arrival	None	n/a	31	Scene (100%)	Blunt: 94% Penetrating: 6%	29 (range 2‐75)
Raitt^62^	Thames Valley, United Kingdom	2014‐2016	To review the appropriateness of PHTRBC and to identify outcomes	Receivers of PHTRBC	None	n/a	n/a	Scene (95%)	Blunt: 53 (84%) Penetrating: 7 (11%) MVC 42 (67%) Fall 8 (13%)	ISS 34 (21‐43)
Sanci	Ontario, Canada	2013–2015	To review blood components transferred with patients from peripheral EDs to a trauma centre	Blood components received at a tertiary care trauma facility	None	n/a	RBC: 127 U in 144 patients	Interfacility (68%)	n/d	n/d
Sunde	Bergen, Norway	2014	To describe our preliminary results after implementing PHTRBC and PHT‐plasma	Receivers of PHTRBC and/or PHT‐plasma	None	n/a	4^b^	Scene (75%)	Blunt: 67% Penetrating: 33%	n/d
Thiels^47^	Minnesota, United States	2002‐2014	To report our experience with pre‐hospital blood product transfusion	Non‐trauma patients receiving PHTRBC and/or PHT‐plasma	Trauma patients receiving PHTRBC and/or PHT‐plasma	No	PHTRBC 654	Scene + interfacility (36%)	n/d	n/d
Wheeler^30^	New England, United States	2005‐2009	To determine factors associated with hypothermia	Trauma patients transported by HEMS, hypothermic on arrival	Non‐hypothermic trauma patients, transported by HEMS	n/a	30	Scene (100%)	n/d	Mean ± SD: Hypothermic: 26.07 ± 11.86 Non‐hypoth: 15.53 ± 11.39
Case reports										
Garner	Sydney, Australia	1997	Case report			n/a	1^b^	Scene (100%)	Blunt: 100%	43 (n = 1)
Lawton	Queensland, Australia	n/s	Case report			n/a	1^b^	Scene (100%)	Blunt: 100%	n/d
Macnab	British Columbia, Canada	1996	Case report			n/a	1	Interfacility (0%)	n/a	n/a
Description of protocol										
Bodnar^24^	Greater Brisbane, Australia	2011–2012	To evaluate the feasibility, limitations and costs involved in PHTRBC	Review of a blood database	n/a	n/a	n/d	n/d (n/d)	n/d	n/d
Escott	Texas, United States	2016‐2017	To address the efficacy, risk and logistical challenges of PHT	n/a	n/a	n/a	23	n/d (20%)	n/d	n/d
Jenkins^59^	Minnesota, United States	2008‐2010	To detail the development and implementation of novel programmes to care for haemorrhage patients requiring PHT	n/a	n/a	n/a	>300	n/d (33%)	n/d	n/d
Lassale^83^	Provence‐Alpes‐Côte d'Azur, France	2010	To present a regional procedure for PHTRBC	n/a	n/a	n/a	n/d	n/d (n/d)	n/d	n/d
Trembley^80^	Minnesota Wisconsin, United States	2016	Description of implementation of protocol	n/a	n/a	n/a	n/d	Scene + interfacility (n/d)	n/d	n/d
Vartanian^81^	Texas, United States	2016	Description of implementation of protocol	Receivers of PHTRBC and/or PHT‐plasma	None	n/a	12	n/d (67%)	Blunt: 7 (87%) Penetrating: 1 (12%) MVC: 5 (62%) Fall: 1 (8%)	n/d
Zielinski^27^	Norway	2016	To disseminate the lessons learned from trauma and haemostasis oxygenation research (THOR) network meeting	Description of PHTRBC in Norwegian HEMS	n/a	n/a	1–2%	Scene + interfacility (n/d)	n/d	n/d
	Minnesota, United States	2016		Description of PHTRBC	n/a	n/a	n/d	Scene + interfacility (n/d)	n/d	n/d
Surveys/forum										
Gillon^37^	Western Australia	2009‐2010	Letter to editor. Mentions international analysis of major haemorrhage management by aeromedical services	Receivers of PHTRBC	none	n/a	58^b^		n/d	n/d
Karl^38^	United States	2012	To characterise the blood‐carrying practices by HEMS programmes across the United States	Surveys to 261 US HEMS programmes	n/a	n/a	n/d	n/d (n/d)	n/d	n/d
Naumann^39^	United Kingdom	2011–2015	To determine how often and which pre‐hospital resuscitation fluids are delivered in the United Kingdom in hypotensive trauma	Patients with hypotensive trauma attended by a doctor in pre‐hospital setting	None	n/a	PHTRBC: 16; 1 of whom also PHT‐plasma	n/d (100%)	Overall hypotensive trauma: Blunt: 654 (92.5%) Penetrating: 53 (7.5%) MVC: 453 (62%) Fall: 92 (13%) Amputation: 2 (0.3%)	n/d
Vardon^36^	France	2012–2013	To survey the means available in the 370 French SMUR for haemorrhagic situations	Survey by email and then phone with all French SMUR leaders	n/a	n/a	n/d	n/d (n/d)	n/d	n/d
Yazer^40^	Australia, Canada, Denmark, France, Germany, Israel, The Netherlands, New Zealand, United Kingdom and United States	2018	To discover how different centres around the world are using blood products and pharmaceuticals in the pre‐hospital setting	International forum	n/a	n/a	n/d	n/d	n/d	n/d
*Military services*										
Prospective comparative studies										
Vitalis^57^	French Armed Forces, Sahel	2016–2017	To evaluate the practices of battlefield transfusion (RBCs, plasma, FWB)	Severely injured receivers of PHT‐RBC or ‐plasma or ‐FWB	No battlefield transfusion	No	7^b^ (4 of whom RBC)	POI + Role 1	Overall: Blunt: 1 (4%) Penetrating: 27 (96%) Explosion 16 (57%) Active external haemorrhage 12 (43%)	PHT: 45 (33‐52) Control: 25 (16‐22) *P* = .01
Retrospective comparative studies										
Howard^35^	US Military, Afghanistan	2001‐2014	To evaluate potential influences on KIA mortality	Casualties who needed and received PHT	Casualties who needed but did not receive PHT	Adjusted data	75^c^	Pre‐hospital helicopter transport to FST or CSH	Overall: Explosion: 65.1% Gunshot: 22.5% Blunt or other: 11.4%	n/d
O'Reilly^55^	UK MERT‐E, Afghanistan	2006‐2011	To evaluate the effect of PHTRBC/PHT‐plasma on mortality	Receivers of PHTRBC and PHT‐plasma	Matched patients where no PHT available	Propensity score	97^b^	POI + Role 1	PHT: Blunt: 1% Penetrating: 99% Burn: 0% Explosive: 51.5% Gunshot wound: 47.4% Matched control: Blunt: 3.1% Penetrating: 96.9% Burn: 0% Explosive: 49.5% Gunshot wound: 47.4%	PHT: 16 (9–25) Control: 16 (9‐24.5) *P* = .686
Shackelford^78^	UK MERT, US Air Force Pedro, US DUSTOFF, Afghanistan	2012‐2015	To examine the association of PHTRBC and/or PHT‐plasma and time to initial transfusion with injury survival	Receivers of PHTRBC and/or PHT‐plasma	No PHT	Frequency matched	55^b^	POI to role 2 or 3	PHT: Explosives 84% Gunshot wound 16% ≥1 amputation: 73% Haemorrhagic torso injury 56% Control: Explosives: 71% Gunshot wound: 29% *P* = .05 ≥1 amputation: 27% *P* < .001 Haemorrhagic torso injury: 35% *P* = .004	PHT: 29 (17–36) Control: 28.6 (24.0‐33.2) *P* = .88
Prospective not‐comparative studies										
Aye Maung^77^	UK Army, Afghanistan	2012‐2014	To explore the utility and feasibility of forward transfusion of RBC	Missions where blood components were carried	None	n/a	2	POI + role 1	Gunshot wound: 100% (n = 2)	n/d
Malsby	US Army, Afghanistan	2012	Process improvement initiative of blood product transfusion on urgent helicopter evacuation casualties	Receivers of PHTRBC and/or PHT‐plasma	None	n/a	15^b^	POI + Role 1	Explosion: 87% Gunshot wound: 13% ≥1 amputation: 60%	n/d
Retrospective not‐comparative studies										
Barkana^76^	Israel Defense Force Medical Corps, Israel	1994‐1996	To characterise the different aspects of PHTRBC and to evaluate its potential effect on the morbidity and mortality	Receivers of PHTRBC	None	n/a	40	POI + role 1	Blunt: 22.5% Penetrating: 77.5% Explosion: 47.5% Gunshot wounds: 22.5% Explosion + gunshot wounds: 7.5% MVC: 20% Fall from height: 2.5%	18 (11.5‐25)
Chen^56^	Israeli Air Force, Israel	2003–2010	To describe PHTRBC, and to evaluate adherence to clinical practice guidelines	Receivers of PHTRBC	None	n/a	89	Scene + interfacility	Combat: 69% Non‐combat: 31% Gunshot wounds: 36% MVC: 28% Explosions: 24% Stab wound: 4% Plane crash: 2% Fall from height: 2%	n/d
Edgar^34^	US and UK Military, Afghanistan	2011	To compare initial management and early outcomes in patients suffering bilateral lower limb amputations and differences related to the type of aeromedical evacuation assets	Surviving adult male patients with bilateral traumatic lower limb amputations transferred by MERT in a CH‐47 Chinook helicopter	Against those transferred by PEDRO in an HH‐60 Pavehawk helicopter	n/a	n/d	POI to Role 3	Only patients with bilateral lower limb amputations	NISS MERT: 27 (range 19–41) PEDRO: 27 (range 22‐29) *P* = 1
Morrison^33^	US and UK Military, Afghanistan	2008–2011	To characterise and compare mortality among casualties evacuated with conventional military retrieval (CMR) to those evacuated with an advanced medical retrieval (AMR) capability	Casualties evacuated from POI by an AMR capability	Against those evacuated by a medic‐led CMR capability	n/a	162^b^	POI to role 3	AMR: Blast: 70.4% Gunshot: 24.3% Other: 5.3% CMR: Blast: 60.8% *P* < .001 Gunshot: 34.9% Other: 4.3%	Mean (SD): AMR: 16 (17) CMR: 15 (16) *P* = .122
O'Reilly^55^	UK MERT‐E, Afghanistan	2008–2011	To present the initial experience of military PHTRBC and PHT‐plasma	Receivers of PHTRBC and/or PHT‐plasma	None	n/a	310^b^	POI to role 2 or 3	Blunt: 1.0% Penetrating: 99% Burn: 0.3% Explosive: 72.9% Gunshot wound 25.8%	20 (16‐29)
Powell‐Dunford^75^	US Army, Afghanistan	2012	To enumerate the specific risks and risk management strategies of en route transfusion	Receivers of PHTRBC and/or PHT‐plasma	None	n/a	61^b^ (54 of whom RBC)	n/d	Explosion: 74% Gunshot wound 26%	n/d
Shlaifer^89^	Israeli Defense Forces, Israel	2013‐2016	To describe feasibility, safety, adverse reactions and adherence to clinical practice guidelines in PHT‐plasma	Receivers of PHT‐plasma. Among them 9 receivers of PHTRBC	None	n/a	9^b^	POI + Role 1	Penetrating: 68.5% Blunt: 15.2% Burn: 1.1% Blast: 1.1% Combination: 14.1%	ISS 1–8:10.9% ISS 9‐14:20.7% ISS 16‐24:28.3% 25‐75:40.1%
Case reports										
West^90^	US Army, Afghanistan	2004	Case report			n/a	1	POI to FST	Penetrating: 100% (n = 1)	n/d

*Note:* Median (IQR) unless otherwise specified.

Abbreviations: AMR, advanced medical retrieval; CC, clinically evident coagulopathic bleeding; CMR, conventional military retrieval; CSH, Combat Support Hospital; FFP, fresh‐frozen plasma; FST, forward surgical team; FWB, fresh whole blood; GI, gastrointestinal; HEMS, Helicopter Emergency Medical Service; ISS, Injury Severity Score; KIA, killed in action; MAST, medical antishock trousers; MERT(−E): Medical Emergency Response Team (−enhanced); MVC, motor vehicle collision; n/a, not applicable; n/d, not described; n/s, not specified for PHTRBC; (N)ISS, (New) Injury Severity Score; PHT, pre‐hospital transfusion; PHTRBC, pre‐hospital transfusion of red blood cells; PHT‐plasma, pre‐hospital transfusion of plasma; POI, point of injury; RBCs, red blood cells; RCT, randomised clinical trial; RTS, revised trauma score; SMUR, Service Mobile d'Urgence et Reanimation (Mobile Emergency and Resuscitation Service); TIC, trauma‐induced coagulopathy; U, Units.

a PHTRBC unless otherwise specified; matched number of patients if applicable.

b PHTRBC and/or other pre‐hospital blood component products.

c Blood products not specified.

We included 14 articles discussing PHTRBC in military medical services. Three articles reported pre‐hospital transfusion as an additional topic, while primarily discussing another aspect of their study^33^, ^34^, ^35^ (Table 1).

### 3.2 *Availability of PHTRBC in civilian services*

Four articles report surveys on the availability of pre‐hospital transfusion to civilian EMS. In France, 84% of 150 responding Mobile Emergency and Resuscitation Services were able to transfuse their patients with RBCs during the mission. However, the survey did not specify whether teams carried the blood products themselves, or had to order them to be delivered to the scene of injury.^36^ Gillon^37^ describes the Australian responses to an international survey of aeromedical services. Three out of seven services had immediate access to PHTRBC; the other four needed 45 minutes in order to obtain it. Karl et al^38^ report that 25.3% of 235 US Helicopter Emergency Medical Service (HEMS) programmes that responded to a survey carried blood, while a 2016 survey found that 10 out of the 22 HEMS in the United Kingdom carried blood products.^39^

An international forum held in 2018 revealed that PHTRBC is practised in Australia, Canada, Denmark, France, Israel, the Netherlands, the United Kingdom, and the United States, and experience with this practice varied from <7 years up to 35 years.^40^ The type of packed cells varied; five out of eight sites provide only O**−** RBCs, two provide only O+ RBCs, and one provides both O+ and O**−** RBCs. One site did not use leuco‐reduced RBCs, and one site used cytomegalovirus‐negative RBCs.

### 3.3 *Frequency of pre‐hospital transfusions*

For civilian EMS that transfuse blood prior to hospital arrival, their overall pre‐hospital transfusion rates vary from 0.2 to 4.4% of patients,^27^, ^41^, ^42^, ^43^, ^44^, ^45^, ^46^, ^47^ without evident differences between interfacility transport and primary transport from the scene (0.7‐6.2%^37^, ^38^ vs 4.9^38^). For the overall population of trauma patients, transfusion rates ranging from 3.0% to 3.5% were reported^48^, ^49^ with transfusion occurring in 1.6% to 7.5% of trauma scene transports^48^, ^49^, ^50^, ^51^ and in 4.8% to 30% of interfacility transports.^48^, ^49^ Cohorts with more severely injured patients (median ISS 22,^25^ trauma score < 12^28^ or “highest risk population”^52^) report transfusions being performed for 19% to 24% of patients.^25^, ^28^, ^52^ In two articles reporting on paediatric patients only, the incidence of transfusion was 28 per 3957 (0.7%) transports,^53^ and of 1176 trauma patients, 16 (1.4%) were transfused.^54^

In military services, pre‐hospital transfusion rates for trauma are markedly higher compiling all patients in civilian EMS, ranging from 14.8% to 26.9% of transports with blood available.^33^, ^55^, ^56^ With regard to only severely injured patients (ISS ≥51^33^ or patient “category alpha”^57^), data are more comparable to the civilian setting, that is, 25% to 32% of more severely injured patients were transfused^33^, ^57^ vs 19% to 24% in the civilian setting.

### 3.4 *Number of units transfused*

In patients treated by a civilian EMS, 5 studies reported a mean/median of 1 unit transfused,^25^, ^29^, ^48^, ^52^, ^58^ 17 studies report a mean/median of 1.1 to 2 units (or between 280 and 560 mL)^37^, ^41^, ^44^, ^45^, ^47^, ^49^, ^51^, ^59^, ^60^, ^61^, ^62^, ^63^, ^64^, ^65^, ^66^, ^67^, ^68^ and 8 studies reported a mean/median of 2.3 to 3 units transfused (or between 560 and 840 mL).^29^, ^31^, ^69^, ^70^, ^71^, ^72^, ^73^, ^74^ Paediatric patients received a mean of 9.8 mL/kg^53^ or 255 mL (8 mL/kg).^54^

In patients treated by a military EMS, most studies (6) reported a mean or median number of PHTRBC transfused of 0.9 to 1,^55^, ^57^, ^75^, ^76^, ^77^, ^78^ one study reported a median of 450 mL^56^ and one study reported transfusion of a median of 4 U of RBCs.^34^

### 3.5 *Transfusion criteria in civilian services*

**Figure 2:**
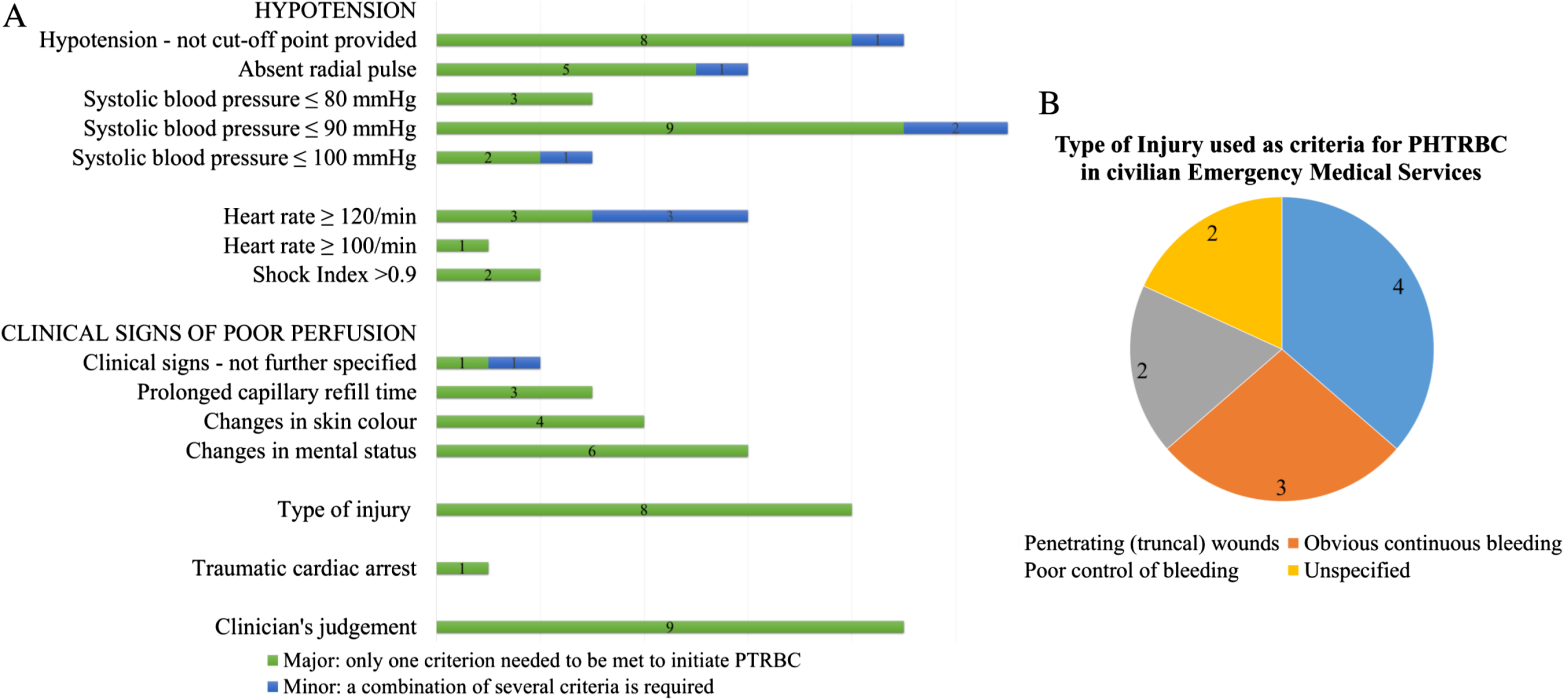
Criteria for pre‐hospital transfusion of red blood cells (PHTRBC) most commonly used in civilian emergency medical services, and injury characteristics used as criteria for PHTRBC in civilian emergency medical services

Figure 2 shows the most common criteria for PHTRBC in adults by civilian services as reported by 20 unique groups. The most commonly reported criteria are follows: hypotension, tachycardia, clinical signs of poor perfusion, type of injury (ie, ongoing bleeding or penetrating wounds) or a clinician's judgement.^25^, ^27^, ^30^, ^41^, ^43^, ^44^, ^45^, ^46^, ^50^, ^51^, ^58^, ^61^, ^64^, ^68^, ^70^, ^73^, ^79^, ^80^, ^81^, ^82^ Not all groups explained their definition of “hypotension” or described which clinical signs were considered to indicate a state of poor perfusion. In Figure 2, these groups have been summarised in the sections “Hypotension—no cut‐off point provided”, and “Clinical signs of poor perfusion—not further specified.” In services where clinicians were explicitly allowed to initiate transfusion based on their clinical judgement, regardless of whether other criteria were met, this is summarised under “Clinician's judgement.”

Some groups used laboratory or point‐of‐care test (POCT) results to aid in making the decision to transfuse (lactate ≥4 mmol/L^46^ or ≥5 mg/dL, INR ≥1.5, base deficit ≥5 mmol/L^79^ or Hb (cut‐off point <9 g/dL, <8 g/dL or < 7 g/dL depending on symptoms and comorbidity.^44^, ^80^ For two groups, ultrasound contributed to decision making (one major, one minor criterion).^25^, ^70^ Furthermore, one group used tissue oxygen saturation (StO_2_) of ≤65% as an indication to transfuse (minor criterion).^79^

In the case of interfacility transport, three groups explained that they would continue a transfusion started by the referring hospital (a major criterion),^43^, ^46^, ^73^ and one group used a haemoglobin concentration reported by the referring hospital as a transfusion trigger (also a major criterion).^73^ Interestingly, 10 groups required the administration of crystalloids before their criteria would prompt transfusion.^30^, ^41^, ^43^, ^44^, ^45^, ^58^, ^61^, ^64^, ^70^, ^73^

### 3.6 *Transfusion criteria for paediatric patients in civilian services*

The criteria used for PHTRBC in children were described by seven groups. Six of these required signs of hypoperfusion to persist after other fluids were administered.^41^, ^45^, ^53^, ^58^, ^73^, ^81^ Five groups specified the required amount as 1 to 3 times 20 to 30 mL/kg of crystalloids before commencing RBCs; Fahy et al had the alternative option of starting with 10 mL/kg of plasma.^53^ The signs of hypoperfusion were described differently in each study: four groups looked at age‐appropriate hypotension,^41^, ^58^, ^73^, ^81^ two groups also examined tachycardia,^45^, ^81^ three groups at “clinical signs of shock”^41^, ^45^, ^53^ and one group transfused if there was only a central pulse.^51^ Additionally, three groups allowed a clinician's judgement to supersede protocol.^41^, ^53^, ^58^

### 3.7 *Transfusion criteria in military services*

Articles were available about pre‐hospital transfusion practices of the armed forces of France, Israel, the United Kingdom and the United States. Only the most recent description of transfusion criteria was included.^35^, ^55^, ^56^, ^57^

**Figure 3:**
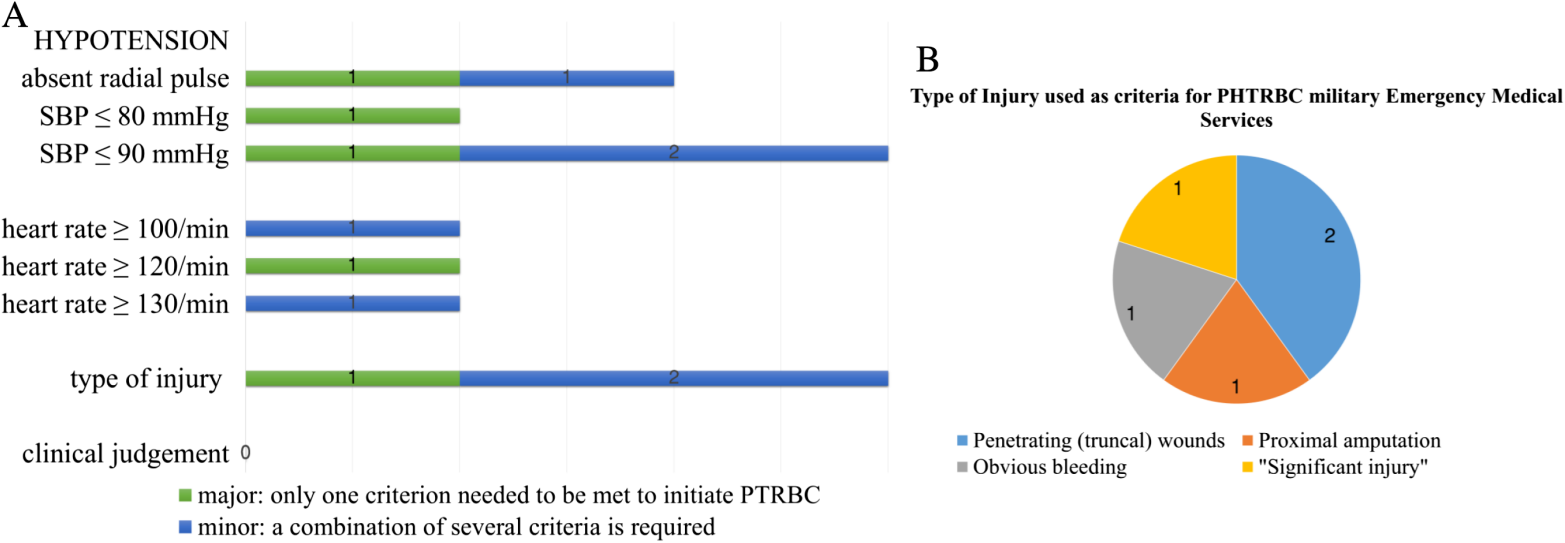
Criteria for pre‐hospital transfusion of red blood cells (PHTRBC) most commonly used in military emergency medical services, and injury characteristics used as criteria for PHTRBC in military emergency medical services

Systolic blood pressure (SBP) <90 mmHg is most often used as the cut‐off point in hypotension, while there is no consensus regarding the cut‐off point for tachycardia (>100/min and >120/min, respectively). The type of injury considered indicative of requiring a transfusion differs; obvious bleeding, proximal amputation or penetrating wounds are used as the criteria for instigation. Unlike the civilian services, only one of the groups advises the use of other fluids before blood products, and none specifically allows a clinician's judgement to supersede protocol (Figure 3). Chen et al^56^ describe the criteria for paediatric patients in the military setting. If there was evidence of persistent “haemorrhagic shock” after 2 × 20 mL/kg of crystalloids, RBCs could then be transfused.

### 3.8 *Challenges of PHTRBC*

Several studies report the difficulties encountered when introducing PHTRBC. In the survey by Karl et al, 5.8% of EMS programmes had discontinued their PHTRBC programme because of access limitations, problematic storage logistics, underutilisation and costs.^38^

Besides requiring a safe transport of blood products, a system needs to be set up to ensure traceability, in cases in which the patient's identity in unknown. It is important that the casualty's blood is drawn for future cross typing prior to transfusion. To minimise wastage, the return of unused units needs to be arranged.^83^

Blood products require a separate intravenous line, and especially in the case of short transport times, transfusion can be performed at the expense of the administration of drugs. Jenkins et al mentioned that for these reasons, after the introduction of pre‐hospital blood products in their service, less patients received tranexamic acid.^59^

Services with a large geographical population and longer transport times have a higher transfusion rate.^45^ For such services, carrying pre‐hospital blood products may be more cost effective. However, evidence regarding cost effectiveness is lacking.

## 4 DISCUSSION

This scoping review identified several studies reporting on the implementation of PHTRBC programmes or pre‐hospital transfusion criteria.

Bleeding patients may require transfusion of RBCs to restore compromised tissue perfusion and oxygenation. Although hard evidence for the beneficial effects of pre‐hospital transfusion is lacking, such transfusions should theoretically improve outcomes in selected patients with substantial blood loss. On the other hand, unnecessary transfusion involves unnecessary risks^84^, ^85^, ^86^ and wastage of resources, in particular, of valuable O negative units. Careful selection of patients is therefore paramount.

Theoretically, the patients to benefit most from early transfusion would be the patients in profound haemorrhagic shock or with ongoing massive bleeding. In hospital, these patients would often trigger the use of a Massive Transfusion Protocol, where massive transfusion is classically defined as the use of 10 or more units of PRBC in the first 24 hours. Also for in‐hospital transfusion, predicting the need for (massive) transfusion has its challenges, and multiple scores have been developed to this end. Each of these scores has limitations, as most of them have been derived retrospectively, were validated in a single centre and may not be applicable to different patient populations; some of them depend on the availability of laboratory and/or ultrasound.^87^ Even more so, it is challenging to appropriately identify patients who require blood transfusions in the pre‐hospital setting, where resources are limited and larger amounts of blood are not readily available.

**Figure 4:**
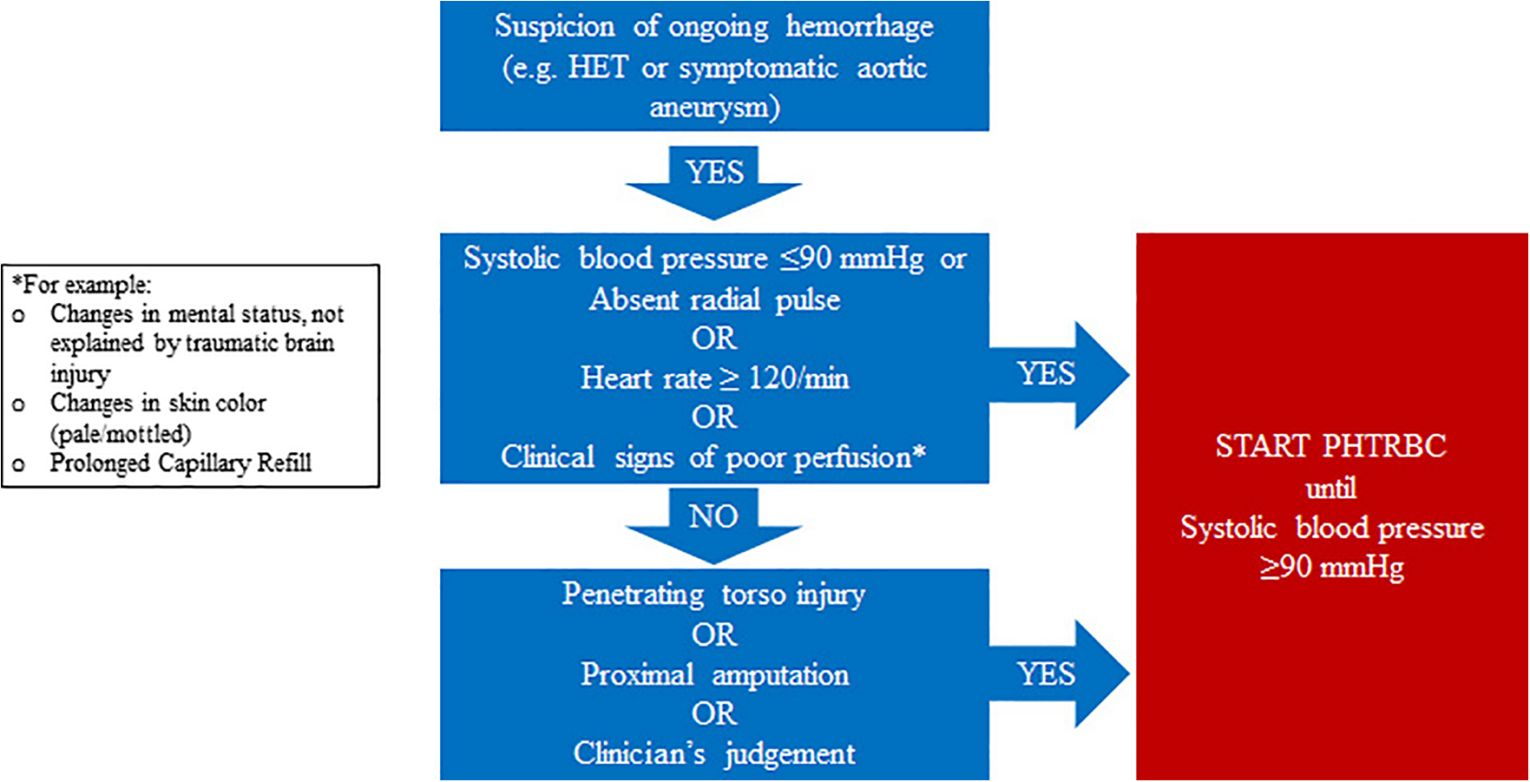
Protocol for the initiation of pre‐hospital transfusion of red blood cells, Helicopter Emergency Medical Service “Lifeliner 1,” Amsterdam, the Netherlands

To the best of our knowledge, pre‐hospital transfusion criteria have not been previously reviewed. Our review does not allow hard conclusions on which patients benefit from PHTRBC. However, we gathered published criteria for PHTRBC that are internationally used. Our systematic review can be considered a cross‐section of current practice regarding indications to initiate PHTRBC. In the absence of evidence, expert opinion is currently the best that is available to guide pre‐hospital transfusion, and our review may aid clinicians to design or refine protocols regarding pre‐hospital transfusion. The protocol for the Amsterdam HEMS has been based on the results of this review, and it is shown in Figure 4.

Our review suggests that there is a broad consensus that hypotension (most often defined as a systolic blood pressure < 90 mmHg), tachycardia (most often defined as a heart rate > 120 beats/min), clinical signs of poor perfusion (in particular changes in mental status) and the type of injury (in particular suspected or confirmed ongoing haemorrhage) are important transfusion triggers in the civilian setting. Similar criteria were reported in military literature.

However, we believe that the decision to initiate or withhold transfusion should not only be based on “hard” criteria but should also involve the clinical judgement of pre‐hospital healthcare providers. For example, a heart rate > 120/min may be a stress response to pain, and changes in mental status are may be due to traumatic brain injury rather than haemorrhage. Such cases should not automatically prompt a transfusion, rather clinical judgement is needed to assess whether transfusion is the most appropriate therapy. In fact, clinical judgement was frequently allowed to supersede the protocol in published literature, and we explicitly agree with this recommendation.

Furthermore, uniform transfusion criteria may ultimately not be optimal: a 20‐year‐old healthy patient is likely able to tolerate a degree of hypotension and tachycardia that an 85‐year‐old is not. Ideally, individual transfusion triggers should be researched. Although not broadly established yet, we suggest studying portable blood‐gas analysers to support well‐informed RBC‐transfusion decisions. Herein, blood‐gas analysis quantifies the markers of anaerobic metabolism during haemorrhage (eg, lactate, pH and base excess) and allows for tracking RBC‐transfusion effects, for example, by restoration of acid/base homeostasis or increase in haemoglobin concentration. In a recent study, we were able to demonstrate the feasibility of portable blood‐gas analysis in the pre‐hospital setting, including pre‐hospital RBC transfusion.^88^ However, further studies are required to study how portable blood‐gas analysis may support RBC‐transfusion decision making, for example, by establishing acid/base‐derived trigger values.

This scoping review also highlights several challenges in the implementation of PHTRBC. EMS operators planning to implement pre‐hospital transfusions need to beware of the pitfalls regarding storage and cooling logistics, return of unused units and traceability of blood products. Practical aspects of administration of blood products in the pre‐hospital environment, such as the need for an additional intravenous line, drawing blood samples for cross‐matching or warming of blood products before transfusion, can all be additional challenges in the pre‐hospital setting in which severely injured patients need to be treated with limited resources and limited personnel.

## 5 CONCLUSIONS

PHTRBC is increasingly used by civilian EMS, but a consensus on transfusion criteria is lacking. This scoping review summarises current practice and may provide the basis for consensus‐based pre‐hospital transfusion protocols. Some studies into the effects of PHTRBC on outcomes have been performed, and an overview of this data will be presented in the second part of this systematic review.

## CONFLICT OF INTEREST

The authors have no competing interests.
